# Developing a standardised approach to the aggregation of inpatient episodes into person-based spells in all specialties and psychiatric specialties

**DOI:** 10.1186/s12911-019-0953-2

**Published:** 2019-11-29

**Authors:** Sarah Rees, Ashley Akbari, Huw Collins, Sze Chim Lee, Amanda Marchant, Arfon Rees, Daniel Thayer, Ting Wang, Sophie Wood, Ann John

**Affiliations:** 1 SAIL Databank, Swansea University Medical School, Swansea, SA2 8PP UK; 2 Health Data Research UK (HDR UK), Swansea University Medical School, Swansea, SA2 8PP UK; 3 Population Psychiatry, Suicide and Informatics, Swansea University Medical School, Swansea, SA2 8PP UK

**Keywords:** Data linkage, Electronic healthcare record, Data quality, Routine health data, Hospital inpatient episodes, Spells

## Abstract

**Background:**

Electronic health record (EHR) data are available for research in all UK nations and cross-nation comparative studies are becoming more common. All UK inpatient EHRs are based around episodes, but episode-based analysis may not sufficiently capture the patient journey. There is no UK-wide method for aggregating episodes into standardised person-based spells. This study identifies two data quality issues affecting the creation of person-based spells, and tests four methods to create these spells, for implementation across all UK nations.

**Methods:**

Welsh inpatient EHRs from 2013 to 2017 were analysed. Phase one described two data quality issues; transfers of care and episode sequencing. Phase two compared four methods for creating person spells. Measures were mean length of stay (LOS, expressed in days) and number of episodes per person spell for each method.

**Results:**

3.5% of total admissions were transfers-in and 3.1% of total discharges were transfers-out. 68.7% of total transfers-in and 48.7% of psychiatric transfers-in had an identifiable preceding transfer-out, and 78.2% of total transfers-out and 59.0% of psychiatric transfers-out had an identifiable subsequent transfer-in. 0.2% of total episodes and 4.0% of psychiatric episodes overlapped with at least one other episode of any specialty.

Method one (no evidence of transfer required; overlapping episodes grouped together) resulted in the longest mean LOS (4.0 days for all specialties; 48.5 days for psychiatric specialties) and the fewest single episode person spells (82.4% of all specialties; 69.7% for psychiatric specialties). Method three (evidence of transfer required; overlapping episodes separated) resulted in the shortest mean LOS (3.7 days for all specialties; 45.8 days for psychiatric specialties) and the most single episode person spells; (86.9% for all specialties; 86.3% for psychiatric specialties).

**Conclusions:**

Transfers-in appear better recorded than transfers-out. Transfer coding is incomplete, particularly for psychiatric specialties. The proportion of episodes that overlap is small but psychiatric episodes are disproportionately affected.

The most successful method for grouping episodes into person spells aggregated overlapping episodes and required no evidence of transfer from admission source/method or discharge destination codes. The least successful method treated overlapping episodes as distinct and required transfer coding. The impact of all four methods was greater for psychiatric specialties.

## Background

National Health Service (NHS) electronic health records (EHRs) are generated during hospital inpatient care in all four nations of the UK (England, Wales, Scotland and Northern Ireland). EHRs are available to use for secondary purposes, and offer a valuable research resource [1]. Each of the four UK nations uses different coding systems and standards for the collection, organisation and analysis of these data, but with many common elements [2–5]. All inpatient EHRs in the UK are organised upon the basic unit of the episode; a period under the care of a single consultant and clinical specialty, in one hospital [6–9]. Administrative information about episodes is recorded in the hospital Patient Administration System (PAS), generally by administrative staff [10]. Clinical information is also recorded at episode level; in all UK nations, when an episode ends, the responsible consultant writes clinical notes in a discharge summary. Clinical coding teams then transform the discharge summary into structured, clinically coded data [11].

The analysis of EHR data across the four UK nations is relatively new, but is becoming increasingly common [12–14]. Devolution of responsibility for health since the late 1990s has led to four increasingly divergent systems, offering an ideal setting for the evaluation of differing organisational and ideological approaches to healthcare provision [15]. The development of a minimum dataset across the four nations is a long-term aspiration [15], but in the absence of such a dataset, researchers using EHR data to compare UK nations require methods that maximise consistency and reproducibility. Inpatient EHRs in each UK nation are similar; it is likely that issues identified and solutions developed in one UK nation’s EHR will be generalizable to the others [16].

The relevance of the episode as a unit for analysis has been questioned [17].. Consecutive episodes for the same individual can be aggregated into types of spell. For example, a hospital provider spell (sometimes referred to as an admission) describes one or more consecutive episodes taking place in the same hospital [18]; when an individual’s care transfers from one hospital to another, a new hospital provider spell commences. Analysis based on hospital provider spells is common and is frequently used for comparing activity by provider [19]; it has value when analysing service utilisation for activities such as capacity and demand planning [20]. However, hospital provider spells may not accurately capture the entire patient pathway, particularly where the patient has been treated in more than one hospital, leading to over-estimation of admissions, and under-estimation of length of stay [16].

Diagnosis-related groups (DRGS) permit comparison of inpatient activity across jurisdictions. DRGs group records with similar conditions based on clinical coding [21] and are commonly used for measuring casemix and calculating provider payment rates [22]. In the UK, inpatient health records are grouped into Healthcare Resource Groups (HRGs). DRGs such as the HRG offer a standardised approach to health data analysis, but they are specific to a hospital spell and do not aggregate records across multiple providers.

Records for an individual can be aggregated regardless of provider, where there is evidence that they are connected; these aggregated records are known as person spells [23], Continuous Inpatient Spells (CIPS) [24] or Continuous Inpatient Stays (CIS) [25] . The person spell may be a more appropriate measure for epidemiological studies, as it better reflects the complete patient journey [26].

There is a hierarchical relationship between episodes and spells; hospital provider spells comprise at least one episode, with person spells comprising at least one hospital provider spell. Most person spells contain one hospital provider spell and one episode [27]. Aggregation into person spells need not be at the expense of higher-level granularity. The underlying identifiers which group records at hospital spell and episode level may be retained, and therefore may still permit analysis at these levels. According to NHS business definitions, a patient should not have concurrent episodes [6], although in reality this does occur, and researchers need to ensure their study methods address these anomalies.

In the UK there are several existing approaches to the aggregation of episodes into variants of person spell. These approaches all require proximity between end date and subsequent start date (usually a maximum of one or two days) and evidence of transfer of care derived from administrative codes [23, 24, 28–30]. However there is no single method upon which all nations of the UK are agreed. Internationally, collaborative work carried out by the Observational Health Data Sciences and Informatics (OHDSI) has resulted in the Observational Medical Outcomes Partnership (OMOP) Common Data Model [31]. The OMOP model specifies a standard structure into which routine datasets can be transformed. Within this model, single instances of conditions may be grouped where certain criteria are met, including records across different settings such as primary and secondary care [32]. However there have been concerns about loss or distortion of data during such transformation, and in particular about how individual entities are counted where multiple providers are involved [33].

One of the challenges of using routine EHR data for research is uncertainty about data quality. Many factors affect data quality including missing data, duplication of records, inconsistent use of standard coding systems and other anomalies such as episodes that overlap. For example, where transfers have taken place it should be possible to identify a pair of records that form the transfer: the transfer-out record and the subsequent transfer-in record. If transfers are not fully captured, the aggregation of episodes into person spells will be affected.

**Fig. 1:**
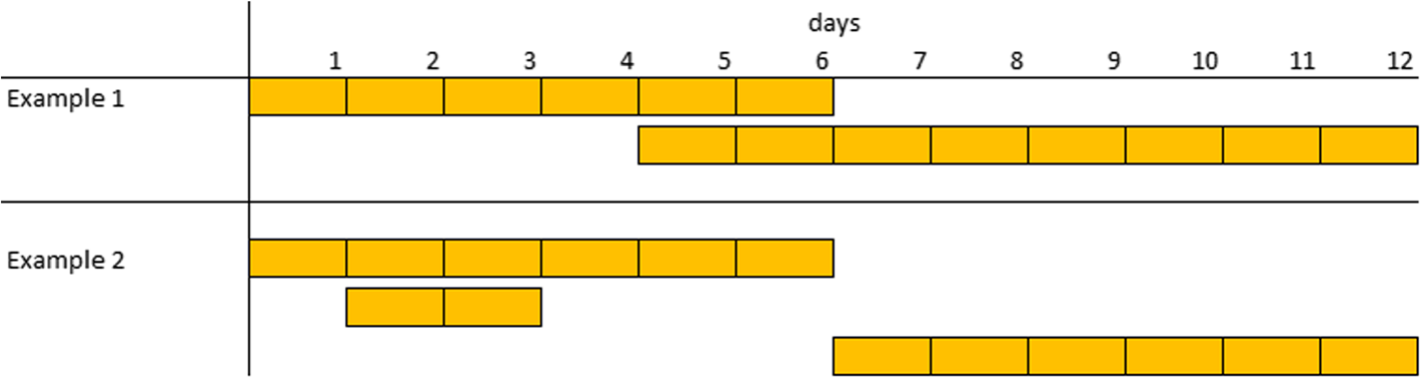
examples of overlapping (Example 1) and nested (Example 2) episodes. Instances where episode sequencing and recording of dates may affect episode aggregation

Anomalies such as overlapping or nested episodes will also affect episode aggregation. Where episodes are grouped based on consecutive ordering, overlapping episodes may not be combined into a single person spell, but rather treated as distinct person spells, as demonstrated in Fig. 1. Example one shows overlapping episodes; depending upon the method use for sequencing episodes, these episodes may be treated as distinct person spells. In example two, there is a negative gap between episodes one and two and a gap greater than one day between episodes two and three. Depending on the aggregation method, these episodes may result in two or possibly three distinct person spells instead of one

In this paper we focus on two elements of data quality important to the aggregation of episodes; coding of transfers of patient care between hospital providers and sequencing of episodes. The study comprised two phases. Phase one described the quality of the data in relation to these elements. Phase two compared four methods for aggregating episodes into person spells. The methods described in this study were developed as part of a wider study of adolescent mental health, which analysed EHRs from all four UK nations [14].

## Methods

### Data source

Hospital inpatient episode data from National Health Service (NHS) providers in Wales were analysed, using extracts held in the Secure Anonymised Information Linkage (SAIL) Databank. The SAIL Databank is a privacy protecting secure research platform holding population level data in Wales, based at Swansea University, which brings together a range of person-level EHR data and enables them to be linked for research purposes [34]. The SAIL Databank permits the linkage of multiple data sources while preserving anonymity by processing data using a split-file approach, with the NHS Wales Informatics Service (NWIS) acting as a Trusted Third Party (TTP). The outputs from this process are pseudo-anonymised data containing an Anonymous Linkage Field (ALF) for each individual that can be successfully matched, alongside associated clinical/event details [35].

The analysis for this study was carried out using the Patient Episode Database for Wales (PEDW). PEDW contains records of all hospital inpatient episodes taking place in NHS healthcare providers in Wales, from the 1990s until the present day, and also includes details about Welsh residents treated in NHS hospitals in England [36]. Episodes where ALF, episode start date, episode end date, admission date or discharge date were null were excluded.

### Data analysis

#### Phase one: assessment of data quality

All hospital provider spells and episodes between 2013 and 2017 (inclusive) were analysed. Transfers-in were analysed using all hospital provider spells with an admission date in the study period, and transfers-out were analysed using all hospital provider spells with a discharge date in the study period. To examine the quality of transfer coding, admissions with a transfer-in code were identified and records were searched to identify whether a preceding admission with a transfer-out code could be identified, where the gap between transfer-out (discharge date) and subsequent transfer-in (admission date) was 0–1 day. Similarly, all discharges ending with a transfer-out code were identified and records searched for a subsequent admission with a transfer-in code, within 0–1 days of discharge. A gap of 0–1 days was chosen as it is common to pre-existing methods in Wales [23] and Scotland [30], and it has been suggested that where times of admission or discharge are not known, a gap of up to 24 h between discharge and subsequent admission is an appropriate means for defining a transfer as opposed to a readmission [37]. Transfers-in and transfers-out were identified using the Admission Method, Admission Source and Discharge Destination codes [4].

Counts of admissions and discharges were calculated by year and specialty group (surgical, medical, psychiatric or other), as were proportions of admissions and discharges where the preceding or subsequent admission or discharge could be identified, either with the criteria that a transfer code was present and there was a gap of 0–1 day, or where the criterion was that there was a gap of 0–1 day only, with no transfer code required.

Date sequencing was analysed by extracting all episodes where the episode start date was between 2013 and 2017 (inclusive). Records were searched to identify all overlapping episodes, all episodes within which another episode was completely nested (referred to as ‘container’ episodes) and all episodes that were completely contained by another episode (referred to as ‘nested’ episodes). Episodes with a zero length of stay were excluded from the sequencing of episodes and resulting analysis, although they were included in the transfer analysis; this was to prevent them being incorrectly counted as overlapping, when they are legitimate episodes. Counts and proportions of overlapping, container and nested episodes were calculated by episode start year and specialty group.

### Phase two: comparison of person spell creation methods

**Fig. 2:**
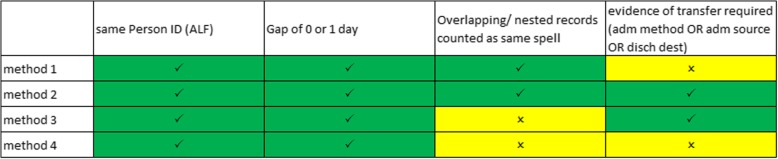
Person Spell definitions. Definitions of the four person spell construction methods that were assessed

All episodes with an admission date (start date of hospital provider spell) between 2013 and 2017 (inclusive) were analysed. This is different to the episode selection criteria in phase one, where episodes were selected based on episode start date, because phase two required inclusion of all episodes within a hospital provider spell, whereas phase one was examining episode overlaps only, with no requirement to link with hospital provider spells. The maximum gap across which to classify episodes as part of the same person spell was set as one day. The four definitions of person spell applied in phase two of the analysis are summarised in Fig. 2. All variants of person spell definition grouped only records where the person identifier (ALF) was the same and where there was a maximum gap of 1 day. Methods 1 and 2 grouped episodes only where there was 0 or 1 day between episodes, and did not group overlapping or nested episodes; methods 3 and 4 grouped all episodes with 1 day or fewer between episodes (distinct from methods 1 and 2 as includes gaps with a negative value), including overlapping or nested episodes. Methods 1 and 4 did not require evidence of transfer between episodes (in the form of transfer codes) whereas methods 2 and 3 grouped episodes only where there was evidence of a transfer. Where transfer codes were a criterion, spells were aggregated where any one of Discharge Destination (from preceding spell), Admission Method or Admission Source (from subsequent spell) indicated a transfer, as described in English [24] and Welsh [23] published methodologies.

As the episode is a common unit of analysis across all four nations in the UK, person spells were created by sequencing episodes rather than hospital provider spells. However as the recording of transfer codes in Wales is at hospital provider spell rather than episode level, the methods involving the use of transfer codes (methods two and three) were applied to hospital provider spell-level data and then joined back to episode-level data to derive the episode counts. SQL stored procedures were created, using DB2 v11.1.4.4, to produce test data outputs for each of the four methods.

Counts of admissions, mean average length of stay and number of episodes per spell were calculated by year for each of the four methods. Data for all specialties and for psychiatric specialties were shown separately. For phase one and two analyses, confidence intervals for proportions were calculated using the Wilson Score Method, and confidence intervals for rates were calculated using Byar’s approximation, as recommended by the Association of Public Health Observatories (APHO) [38].

## Results

### Phase one: assessment of data quality

#### Transfer coding

**Fig. 3:**
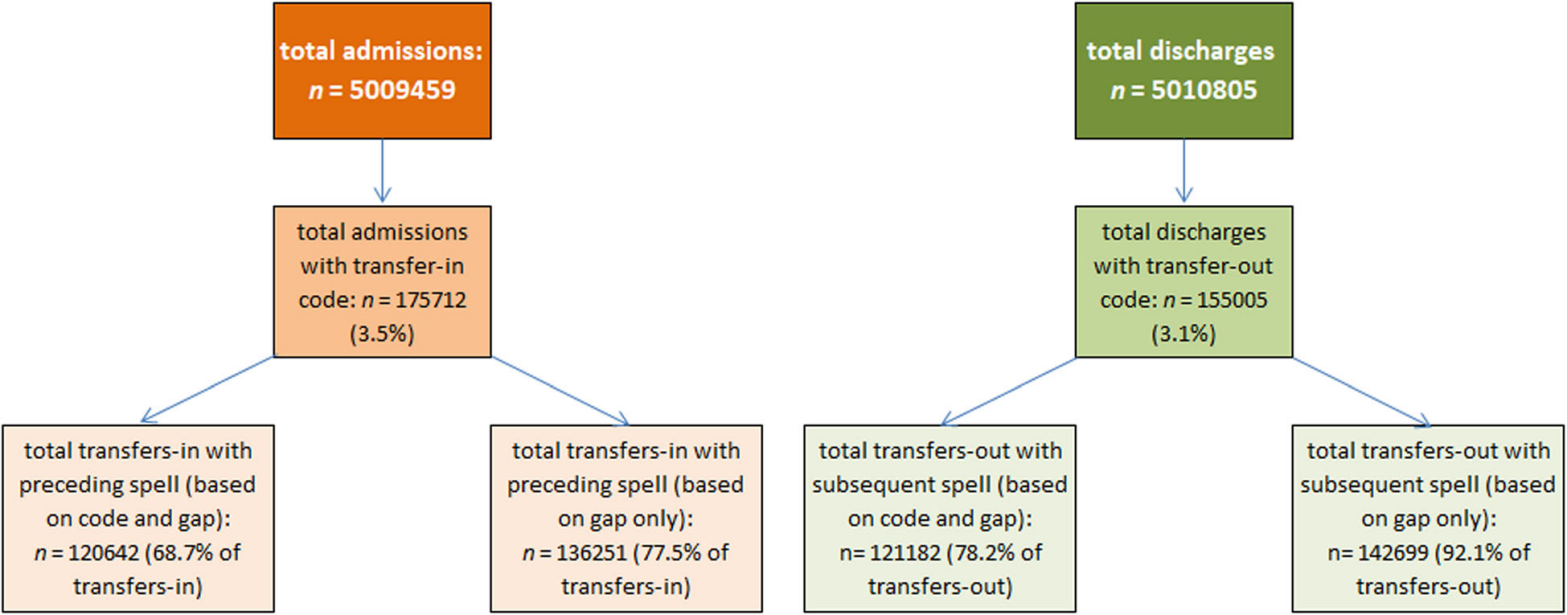
summary of admissions and discharges coded as transfers-in or transfers-out. Total admissions and discharges, subtotals with transfer in/out codes and the proportion of transfers in/out with an identifiable preceding or subsequent transfer episode (with either a relevant transfer code and a gap of 0–1 day, or with a gap of 0–1 day only)

Analysis of transfer coding of Welsh hospital provider spells data where the admission or discharge date was between 2013 and 2017 is summarised in Fig. 3.

**Fig. 4:**
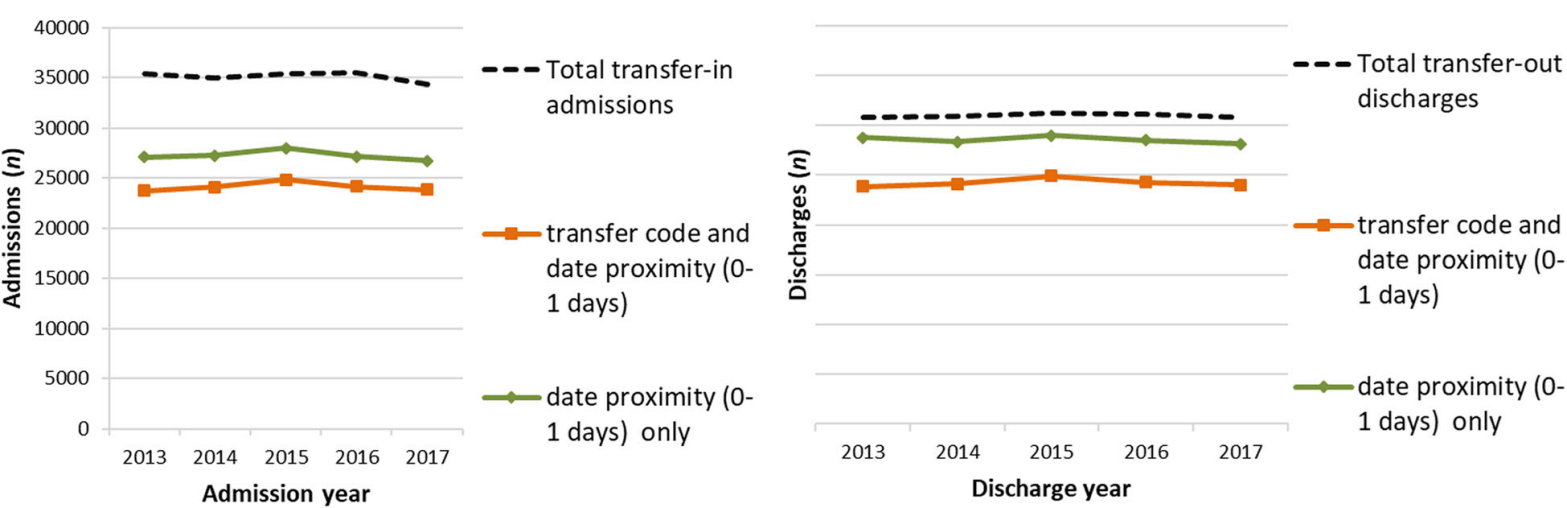
transfers-in with preceding transfer-out and transfers-out with subsequent transfer-in. Summary by admission/discharge year of transfers-in or transfers-out with an identifiable preceding or subsequent transfer episode (with either a relevant transfer code and a gap of 0–1 day, or with a gap of 0–1 day only)

There were 5,009,459 admissions and 5,010,805 discharges between 2013 and 2017. 3.5% (95% CI 3.5%; 3.5%) of total admissions were coded as transfers-in, and 3.1% (95% CI 3.1%; 3.1%) of discharges were coded as transfers-out. Of 175,712 transfers-in, 68.7% (95% CI 68.4%; 68.9%) had an identifiable preceding transfer-out within one day, and of 155,005 transfers-out, 78.2% (95% CI 78.0%; 78.4%) had an identifiable subsequent transfer-in within one day. These proportions rise to 77.5% (95% CI 77.3%; 77.7%) for transfers-in and 92.1% (95% CI 91.9%; 92.2%) for transfers-out when the requirement for a transfer code is removed and hospital spells are grouped purely based on date proximity. Counts and proportions remained stable over the study period, as summarised in Fig. 4.

Analysis by specialty group showed that psychiatric specialties had the highest proportion of transfers-in and transfers-out. Of 54,114 psychiatric admissions, 25.4% (95% CI 25.0%; 25.8%) were transfers-in and of 55,191 psychiatric discharges, 12.4% (95% CI 12.1%; 12.7%) were transfers-out. In comparison of 1,685,410 surgical admissions, 2.0% (95% CI 1.9%; 2.0%) were transfers-in, of 1,677,835 surgical discharges, 2.1% (95% CI 2.1%; 2.1%) were transfers-out, of 1,699,048 medical admissions, 4.3% (95% CI 4.3%; 4.4%) were transfers-in and of 1,662,508 medical discharges, 4.6% (95% CI 4.6%; 4.6%) were transfers-out.

**Fig. 5:**
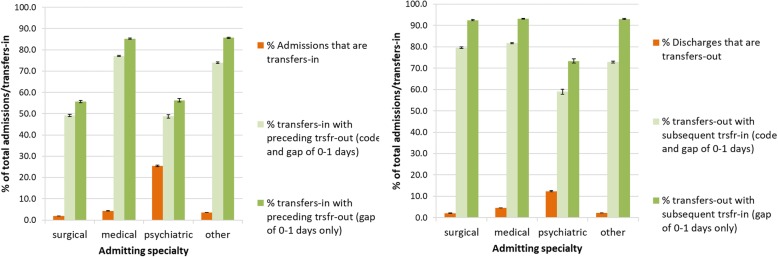
transfers-in and transfers-out and percentage with identifiable preceding transfer-out or subsequent transfer-in, by specialty group. Summary by broad clinical specialty of transfers-in or transfers-out with an identifiable preceding or subsequent transfer episode (with either a relevant transfer code and a gap of 0–1 day, or with a gap of 0–1 day only)

Transfers-in and transfers-out of psychiatric specialties were less likely than other higher-volume specialties to have an identifiable preceding transfer-out or subsequent transfer-in, particularly compared with medical admissions and discharges. Of 13,747 transfer-in admissions into a psychiatric specialty, it was possible to identify a preceding hospital spell (regardless of specialty) with a transfer-out code and a discharge date within one day of the subsequent admission date for 48.7% (95% CI 47.9%; 49.6%), increasing to 56.3% (95% CI 55.5%; 57.1%) when searching for date proximity only. Of 6831 transfer-out discharges from a psychiatric specialty, it was possible to identify a subsequent hospital spell (regardless of specialty), with a transfer-in code and admission date within one day of the preceding discharge date for 59.0% (95% CI 57.8%; 60.1%), increasing to 73.4% (95% CI 72.3%; 74.4%) when searching for date proximity only. Of 33,044 transfer-in surgical admissions, 49.2% (95% CI 48.7%; 49.7%) had an identifiable preceding transfer-out, increasing to 55.7% (95% CI 55.1%; 56.2%) when searching for date proximity only (similar to rates for psychiatric admissions). Of 35,618 transfer-out surgical discharges, 79.6% (95% CI 79.2%; 80.0%) had an identifiable subsequent transfer-in, increasing to 92.4% (95% CI 92.1%; 92.7%) when searching for date proximity only (considerably higher than rates for psychiatric discharges). Of 73,534 transfer-in medical admissions, 77.1% (95% CI 76.8%; 77.4%) had an identifiable preceding transfer-out, increasing to 85.2% (95% CI 85.0%; 85.5%) when searching for date proximity only, and of 76,751 transfer-out medical discharges, 81.7% (95% CI 81.4%; 82.0%) had an identifiable subsequent transfer-in, increasing to 93.1% (95% CI 93.0%; 93.3%) when searching for date proximity only (considerably higher than rates for psychiatric specialties for both transfers-in and transfers-out). These results are summarised in Fig. 5.

#### Episode sequencing

**Fig. 6:**
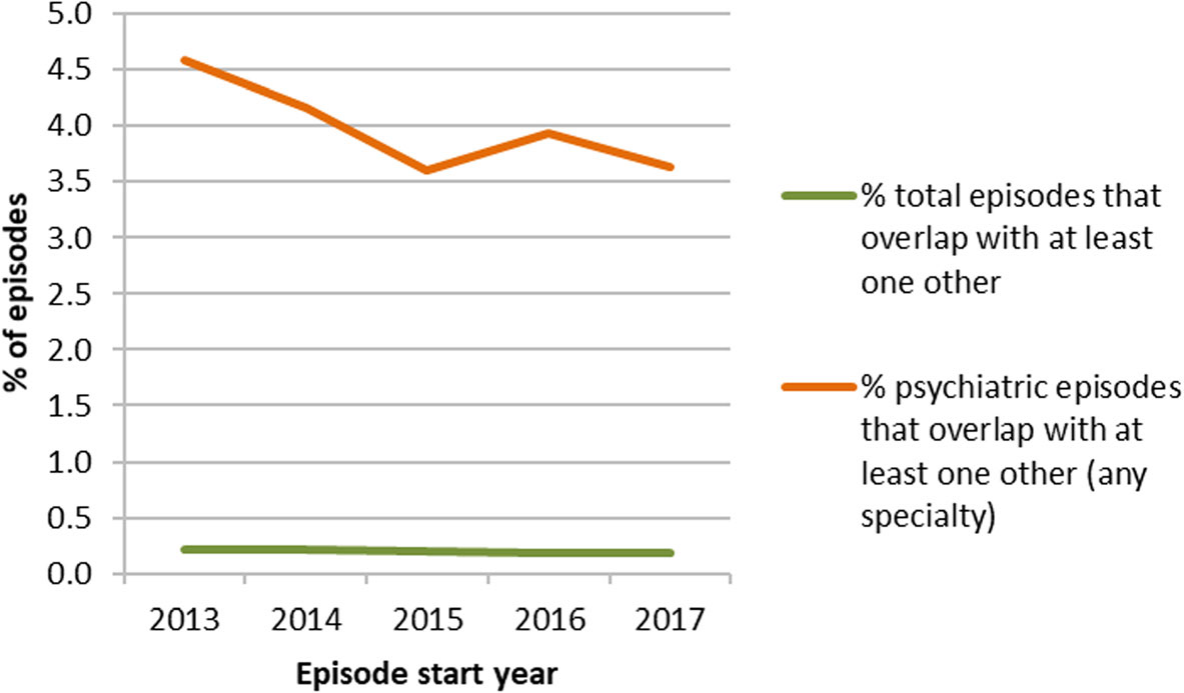
overlapping episodes. Summary by year and specialty (all or psychiatric) of the proportion of episodes that overlap with at least one other episode of any specialty

**Fig. 7:**
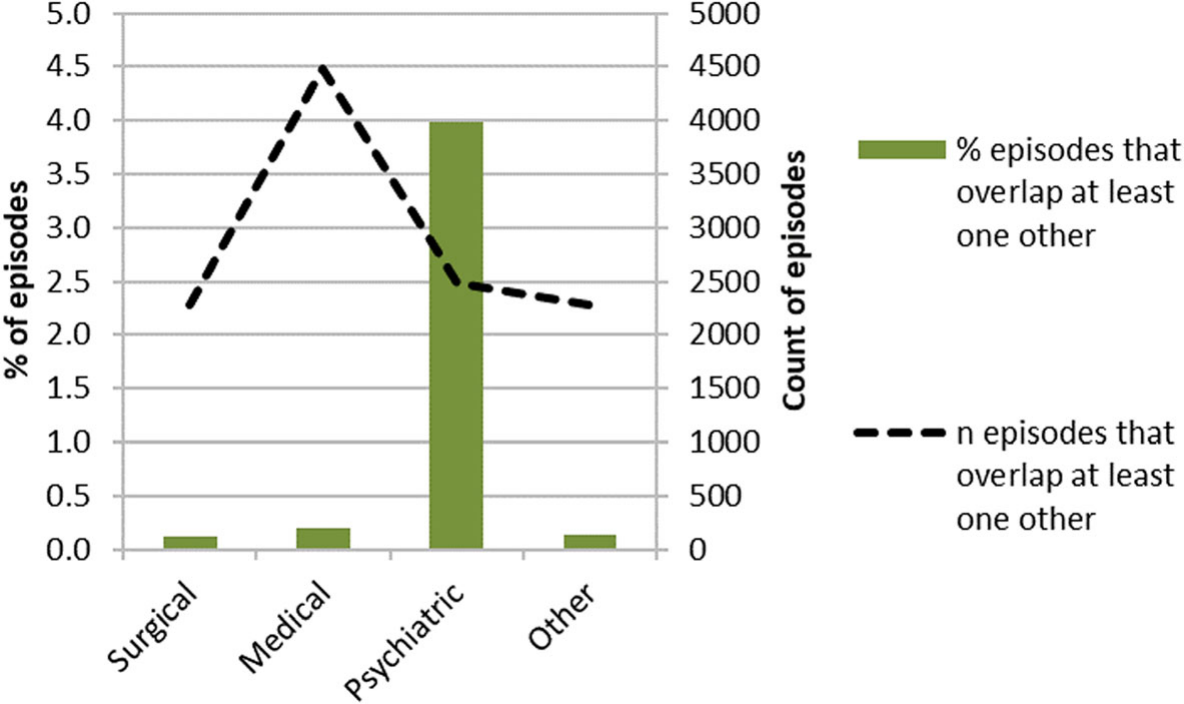
overlapping episodes by specialty. Summary by broad specialty group of the proportion of episodes that overlap with at least one other episode of any specialty

There were 5,920,641 episodes starting between 2013 and 2017. In total, 62,355 episodes (1.1% of total episodes) were under psychiatric specialties, 1,820,198 (30.8% of total episodes) were under surgical specialties, 2,286,044 (38.6% of total episodes) were under medical specialties and 1,750,486 (29.6%) were under other specialties. Of total episodes, 0.2% (95% CI 0.2%; 0.2%) overlapped at least one other episode. Psychiatric specialties had the highest proportion of overlapping episodes; of 62,355 psychiatric episodes, 4.0% (95% CI 3.8%; 4.1%) overlapped with another episode (of any specialty). In comparison, of 2,286,044 medical episodes, 0.2% (95% CI 0.2%; 0.2%) overlapped and of 1,820,198 surgical episodes, 0.1% (95% CI 0.1%; 0.1%) overlapped. Figure 6 summarises overlapping episodes by year, for all specialties and for psychiatric specialties. Of total overlapping episodes, 38.8% (95% CI 37.9%; 39.7%) were under medical specialties, 21.6% (95% CI 20.8%; 22.3%) were under psychiatric specialties and 19.8% (95% CI 19.1%; 20.5%) were under surgical specialties. Figure 7 summarises overlapping episodes by specialty.

Of total episodes, 0.1% (95% CI 0.1%; 0.1%) completely contained at least one other episode (‘container’ episodes); this is a subset of overlapping episodes. The proportion is small but it affects some specialties disproportionately; 3.5% (95% CI 3.4%; 3.6%) of total psychiatric episodes were container episodes and 37.9% (95% CI 36.7%; 39.2%) of all container episodes were under a psychiatric specialty. In comparison 0.1% (95% CI 0.1%; 0.1%) of all medical episodes and < 0.1% (95% CI 0.03%; 0.04%) of all surgical episodes were container episodes, 30.5% (95% CI 29.3%; 31.7%) of container episodes were under medical specialties and 10.9% (95% CI 10.2%; 11.8%) of container episodes were under surgical specialties.

0.2% (95% CI 0.2%; 0.2%) of all episodes were completely contained within another episode (‘contained’ episodes); as with container episodes, this is a subset of overlapping episodes. 50.7% of all contained episodes were under medical specialties, but only 0.3% (95% CI 0.3%; 0.3%) of all medical episodes were contained episodes. In contrast, only 7.2% (95% CI 6.8%; 7.7%) of all contained episodes were under psychiatric specialties, but 1.4% (95% CI 1.3%; 1.5%) of all psychiatric episodes were contained episodes.

### Phase two: comparison of person spell creation methods

Phase one results showed that data quality issues affected psychiatric specialties more than other specialty groups (as shown in Fig. 5, Figure 7); therefore analysis of the results for phase two are shown for all specialties and separately for psychiatric specialties. 5,893,995 episodes commenced between 2013 and 2017 (this is different to the total episodes in phase one, as phase two episodes were selected based on hospital provider spell start date rather than episode start date, to ensure that all episodes within a hospital provider spell were included). Of these episodes, 61,056 (1%) were under psychiatric specialties. Psychiatric specialties comprise Adult Mental Illness (70% of episodes), Old Age Psychiatry (26% of episodes), Child and Adolescent Psychiatry (1% of episodes) and other psychiatric specialties (3% of episodes).

Method one resulted in the fewest person spells; person spell count was 76.1% (95% CI 76.0%; 76.1%) of total episodes, and method three resulted in the most person spells; person spell count was 82.4% (95% CI 82.3%; 82.4%) of total episodes. This means that method one grouped the highest number of episodes together, creating person spells with a higher number of episodes. Person spells created using methods one and four contained 1.3 (95% CI 1.3; 1.3) episodes per person spell, compared with methods two and three which contained 1.2 (95% CI 1.2; 1.2) episodes per person spell. The pattern for person spells containing at least one psychiatric episode was similar; method one person spell count was 78.5% (95% CI 78.2%; 78.8%) of total episodes and method three person spell count was 82.8% (95% CI 82.5%; 83.1%) of total episodes. Person spells containing at least one psychiatric episode created using method one had 1.3 (95% CI 1.3; 1.3) episodes per spell compared with 1.2 (95% CI 1.2; 1.2) episodes for methods two, three and four.

**Fig. 8:**
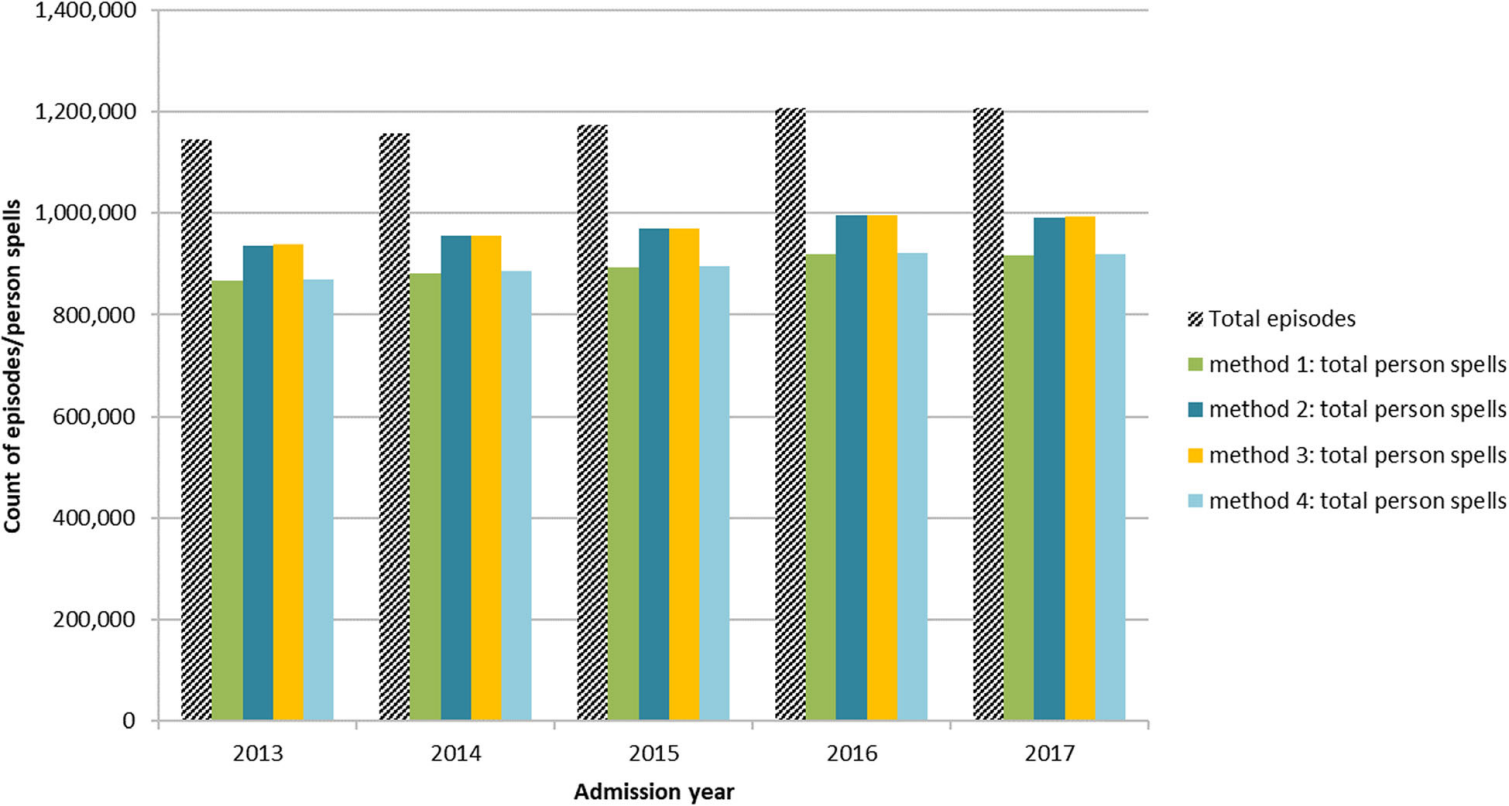
Person spells by method and admission year, as a proportion of total episodes. Summary showing the relationship between total episodes and total person spells resulting from each of the four person spell construction methods, for episodes of all clinical specialty

**Fig. 9:**
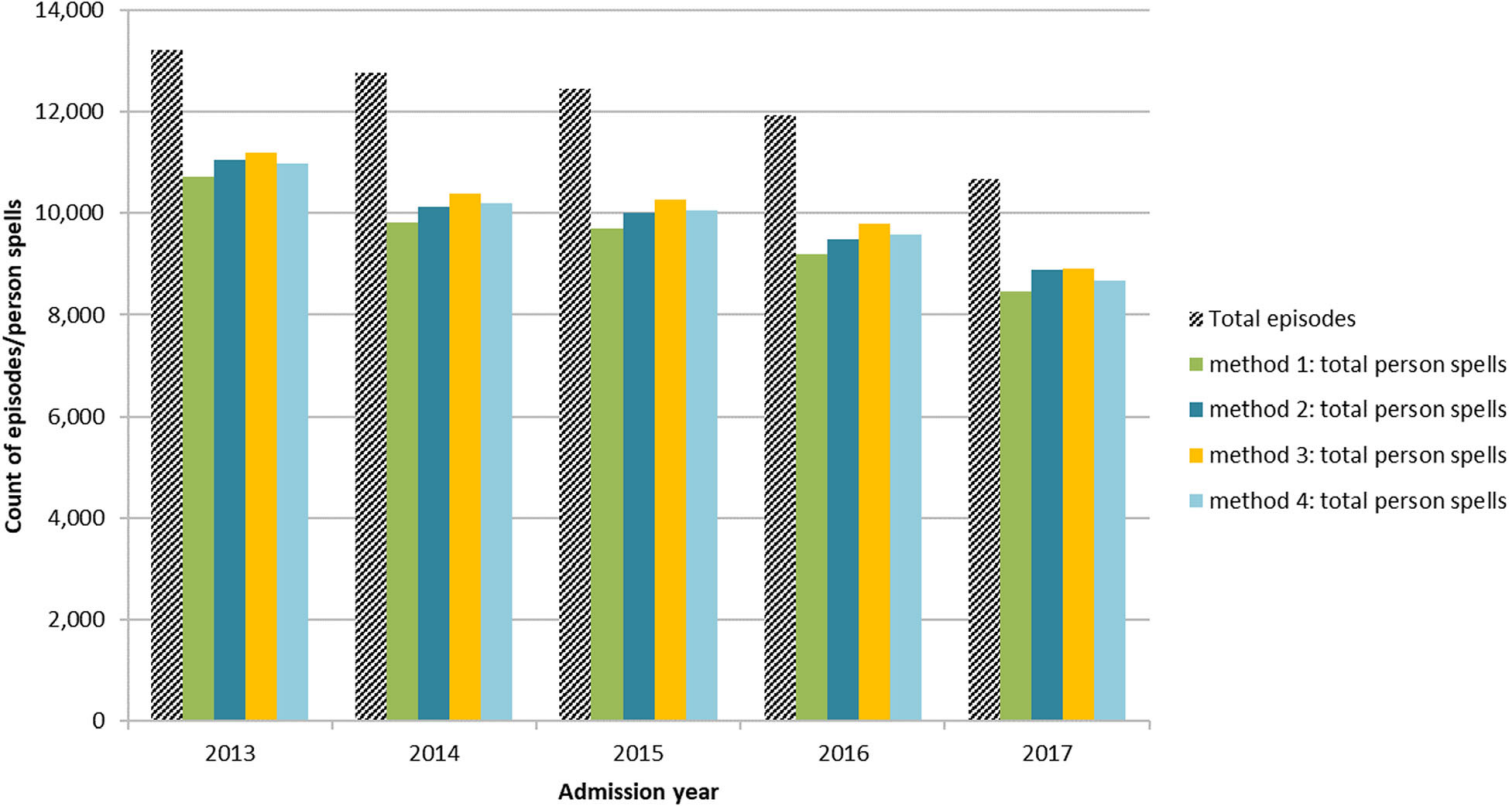
Person spells by method and admission year, as a proportion of total episodes, for person spells containing at least one psychiatric episode. Summary showing the relationship between total episodes and total person spells resulting from each of the four person spell construction methods, for episodes of psychiatric specialty only

Figure 8 and Figure 9 summarise the results by admission year of each method compared with the original number of episodes, for all specialties and for person spells containing at least one episode under psychiatric specialties.

Across all specialties, method one resulted in 82.4% of all person spells containing a single episode only, compared with 86.9% for methods two and three and 82.5% for method four. For person spells containing at least one psychiatric episode, the pattern is slightly different; 69.7% of person spells created by method one were single episode spells, compared with 84.8% by method two, 86.3% by method three and 73.3% by method four.

Table 1 summarises the effect on mean length of stay (LOS) of each of the four methods. All of the methods considerably increased the mean LOS when compared with LOS based on episodes, with method one resulting in the longest mean LOS (4.0 days/SD 18.2 for all specialties and 48.5 days/SD 111.5 for psychiatric specialties) and method three the shortest (3.7 days/SD 17.3 for all specialties and 45.8 days/SD 106.1 for psychiatric specialties). When comparing length of stay derived from episodes and from person spell variants, method one resulted in an increase of 1 day (34.0%) for all specialties and 13.1 days (36.8%) for psychiatric specialties whereas method three increased by 0.7 days (22.3%) for all specialties and 10.3 days (29.1%) for psychiatric specialties.

**Table 1:** Length of stay

		Method 1	Method 2	Method 3	Method 4
Person spells: all specialties	Mean LOS	4.0	3.7	3.7	4.0
	SD LOS	18.2	17.3	17.3	18.4
Person spells: psychiatric specialties	Mean LOS	48.5	46.7	45.8	48.6
	SD LOS	111.5	107.5	106.1	113.1
Results from underlying episodes, so no method for aggregation applied					
Episodes: all specialties	Mean LOS	3.0			
	SD LOS	12.6			
Episodes: psychiatric specialties	Mean LOS	35.5			
	SD LOS	79.1			
Difference (days LOS) between episode and person spell	All specialties	1.0	0.7	0.7	1.0
	psychiatric specialties	13.1	11.3	10.3	13.2
Difference (%) between episode and person spell	All specialties	34.0	22.3	22.3	34.7
	psychiatric specialties	36.8	31.8	29.1	37.1

## Discussion

### Main findings

Previous studies have compared length of stay derived from episodes, hospital provider spells and person spells, arguing that using episodes or hospital provider spells can significantly under-estimate length of stay and potentially over-estimate hospital activity and disease incidence [16, 39]. No studies appear to have examined different methods for constructing person spells, or addressed the impact of data quality on spell construction. This study examined two important aspects of data quality and four methods for spell construction, with the aim of developing a method for the creation of person spells that permits comparative measurement of key indicators both within and between UK nations.

Phase one analysis examined the data quality of key variables used in the creation of person spells. We found gaps in the coding of transfers of care, with transfers-in captured more thoroughly than transfers out. Figure 4 shows that 68.7% of transfers-in had an identifiable preceding transfer-out, and 78.2% of transfers-out had an identifiable subsequent transfer-in (where date proximity and a relevant transfer code were required). The analysis suggests that some transfers are not being captured fully by the transfer coding, as they can be identified by searching for date proximity alone; when the requirement for transfer coding was removed and episodes were linked based only on date proximity, 77.5% of transfers-in had an identifiable preceding transfer-out, and 92.1% of transfers-out had an identifiable subsequent transfer-in. Psychiatric discharges are particularly affected, as shown in Figure 5. Methods which use transfer coding as a necessary criteria for aggregation will fail to link episodes where coding is incomplete.

Although the number of overlapping or nested episodes is small, the proportion of affected records is greater for episodes in psychiatric specialties (as shown in Figs. 6 and 7) and is sufficient to affect spell counts and length of stay calculations. Depending on the method used to sequence episodes, the presence of concurrent episodes may introduce incorrect breaks during what should be a single person spell. Specialties such as psychiatry, which have longer overall lengths of stay, may be particularly affected as they may be more likely to contain nested episodes of care under different specialties, such as when patients have episodes of chemotherapy or dialysis. This may result in failure to aggregate genuinely connected records, leading to inflated spell count and reduced mean length of stay.

These findings are important because incorrect or incomplete transfer coding or episode sequencing may lead to the misclassification of episodes as distinct person spells, inflating the number of person spells, therefore over-estimating disease incidence and prevalence, and under-estimating average lengths of stay.

As shown in Figs. 8 and 9 and Table 1, all methods for spell construction resulted in increased length of stay and reduced spell counts. The most successful method for grouping episodes (the method that linked together the greatest number of episodes, leading to the largest number of episodes per person spell and the smallest number of single-episode person spells) was method one, which grouped together overlapping episodes and did not require the presence of a transfer code. Results obtained using methods one and four were broadly similar. Length of stay for all specialties increased from 3.0 to 4.0 days (34.0%) using method one and from 3.0 to 4.0 days (34.7%) using method four. For psychiatric specialties LOS increased from 35.5 to 48.5 days (36.8%) using method one and from 35.5 to 48.6 days (37.1%) using method four. Results for methods two and three were also similar; LOS for all specialities increased from 3.0 to 3.7 days (22.3%) for both methods two and three. For psychiatric specialties LOS increased from 35.5 to 46.7 days (31.8%) using method three and from 35.5 to 45.8 days (29.1%) using method four. These results suggest that errors in transfer coding have a greater impact on total person spell counts and mean LOS than errors in episode sequencing. Although the effect of episode sequencing is very small overall, it is greater at specialty level, in particular for psychiatric specialties. These findings are useful for research using routine health data, as they identify sources of error in disease incidence estimation and service utilisation such as hospital admissions and length of stay measures.

### Strengths and limitations

The analysis in this study was based on Welsh data only. Lack of access to full datasets from England, Scotland and Northern Ireland meant that we were not able to analyse data from all four UK nations at the level of detail in this study. However it is likely that our findings reflect similar issues with datasets in other UK nations, as the approach to data collection is broadly similar [16]. The person spell methodology we developed was successfully implemented using data from England, Wales, Scotland and Northern Ireland as part of the HQIP Adolescent Mental Health project [14].

The primary aim of the study was to find ways of grouping data that better reflected the patient journey and could be applied to multiple datasets across jurisdictions, to provide a consistent approach when carrying out comparative analysis. As far as the authors are aware, this is the first study to examine the impact of data quality on methods for person spell construction. Analysis using person spells as a basic unit of healthcare should represent measures such as length of stay and number of admissions more accurately than episodes or hospital spells. Grouping episodes together based on date proximity alone may result in the incorrect aggregation of some episodes but it is anticipated that this would be minimal compared with the gains due to greater aggregation of genuinely connected episodes.

Aggregation at person spell level adds an additional level of granularity but not at the expense of higher levels of granularity, as the identifiers for episode and hospital spell are retained in the dataset and therefore support analysis at all levels of granularity.

### Implications for future research

The methods described in this study are generalizable to inpatient data from all four nations of the UK, but it is important to note that as the coding of transfers in Scotland is different, the administrative codes required to identify these records will require harmonisation for consistency across all nations. Further work could replicate the analysis carried out in this study using the data from other UK nations.

The analysis in this study was carried out on all episodes, including day cases and regular admissions (e.g. a planned sequence of admissions for treatments such as chemotherapy or dialysis). It may not always be appropriate to group together all types of admission; for example there are many instances where long-stay psychiatric episodes contain a nested episode for a planned treatment such as chemotherapy or dialysis. It would be useful to assess the impact of regular admissions as these types of admission are more likely to be nested within another episode.

The criteria for the most successful aggregation method was that which grouped together the greatest number of episodes. It is possible that incorrect grouping of clinically distinct episodes will occur. Examination of the clinical coding of linked episodes within each person spell would allow this to be explored.

## Conclusions

There are compelling reasons to group health records into linked sequences of episodes, but across the UK there is no single definitive method for doing so. The aim of this study was to decide the best method for bringing together routine health datasets from each of the four UK nations, to enable consistent comparison of measures such as disease incidence and length of stay, both within and between nations. It describes an approach to the aggregation of hospital inpatient episodes that is applicable to routine health data from all UK nations.

The method that successfully linked the greatest number of episodes (and therefore created the lowest number of person spells) did not require presence of transfer codes and grouped together overlapping as well as consecutive episodes. Researchers should ensure that their analysis considers these factors as they may affect the calculation of important health measures.

As the analysis of cross-UK health data becomes more common, it is hoped that studies such as this will benefit researchers when deciding upon analysis methods. By sharing knowledge and experience about data exploration and methods for analysing EHR data, the research process will become more efficient and duplication of effort can be minimised.

## Data Availability

The datasets generated and analysed during the current study are not publicly available, as the anonymised data held by SAIL can only be accessed in the SAIL secure remote access environment, within the context of an approved project. However, data can be accessed on approval from SAIL and reasonable request to the authors. Data held within the SAIL Databank are made available to researchers in an anonymised format, and are therefore not subject to data protection legislation. SAIL follows all relevant legislative and regulatory frameworks in using population data for research.

## Abbreviations

ALF: Anonymous Linkage Field - a unique patient identifier contained in each SAIL dataset. Used to join people between different datasets
APHO: Association of Public Health Observatories
CEMACH: Confidential Enquiry into Maternal and Child Health
CIP: Continuous Inpatient Spell - English terminology
CIS: Continuous Inpatient Stay - Scottish terminology
CMACE: Centre for Maternal and Child Enquiries
EHR: Electronic Health Record
DRG: Diagnosis-related Group
HQIP: Healthcare Quality Improvement Partnership
HRG: Healthcare Resource Group
IGRP: Information Governance Review Panel
LOS: Length Of Stay
NCEPOD: National Confidential Enquiry into Patient Outcome and Death
NHS: National Health Services
NWIS: NHS Wales Informatics Service
OHDSI: Observational Health Data Sciences and Informatics
OMOP: Observational Medical Outcomes Partnership
PAS: Patient Administration System
PEDW: Patient Episode Database for Wales
SAIL: Secure Anonymised Information Linkage (Databank)
TTP: Trusted Third Party
